# Changes in the urine volatile metabolome throughout growth of transplanted hepatocarcinoma

**DOI:** 10.1038/s41598-022-11818-0

**Published:** 2022-05-11

**Authors:** M. Yu. Kochevalina, A. B. Bukharina, V. G. Trunov, A. V. Pento, O. V. Morozova, G. A. Kogun’, Ya. O. Simanovsky, S. M. Nikiforov, E. I. Rodionova

**Affiliations:** 1 Kharkevich Institute for Information Transmission Problems, Russian Academy of Sciences, Moscow, Russia; 2 Prokhorov General Physics Institute of the Russian Academy of Sciences, Moscow, Russia; 3 N.N. Blokhin National Medical Research Center of Oncology, Ministry of Health of the Russian Federation, Moscow, Russia; 4 Cynological Division of Aviation Security Service, Aeroflot, Russian Airlines, Moscow, Russia

**Keywords:** Cancer, Biomarkers, Oncology, Chemistry

## Abstract

Trained detection dogs distinguish between urine samples from healthy organisms and organisms with malignant tumors, suggesting that the volatile urine metabolome contains information about tumor progression. The aim of this study was to determine whether the stage of tumor growth affects the chemical differences in the urine of mice and to what extent the "olfactory image of disease" perceived by dogs coincides with the "image of disease" recorded by the mass spectrometer. We used a novel laser ionization mass spectrometry method and propose a mass spectrometric analysis without detailed interpretation of the spectrum of volatile metabolomes in urine. The mass spectrometer we use works without sample preparation and registers volatile organic compounds in air at room temperature without changing the pH of the sample, i.e. under conditions similar to those in which dogs solve the same problem. The experimental cancer models were male BDF-f1 hybrid mice transplanted with hepatocarcinoma tissue, and similar mice transplanted with healthy liver tissue were used as controls. Our data show that both dogs and our proposed laser mass spectrometry method are able to detect both the entire spectrum of volatile organic compounds associated with the disease and minor changes in this spectrum during its course.

## Introduction

Volatile organic compounds (VOCs) produced by living organisms during metabolism have recently been studied using various methods^1–5^. They are a highly developed form of chemical communication between organisms and encode information necessary for conspecifics’ regulating behaviour and physiology^6,7^.

Almost every disease or body injury causes a change in metabolism, which leads to a change in the composition or ratio of VOCs. These include helminthic invasions^8,9^, infectious diseases^10–13^immunization^14^, chronic diseases^15^, brain damage^16^, and even minor physical injury, such as from an injection^17^. It has also been shown that VOCs used as tumour markers are highly likely to be present in the excreta of organisms, e.g., samples of exhaled air^18–21^, urine^22,23^, and faeces^23,24^ of human and animals. VOCs can also be detected by instrumental methods, primarily mass spectrometers, and can be used not only as biomarkers for diagnosing various diseases^11,25^ but also for developing rapid screening. It is also important that "volatile markers" of the disease are present in the body's natural secretions and their collection is a non-invasive procedure, which is very important for severely ill patients and young children.

The main issue in the application of VOC analysis for diagnostics is extracting meaningful information, which makes it possible to clearly distinguish sick organisms from healthy ones. The use of instrumental methods makes it possible to isolate a large number of volatile compounds and identify them. Thus, hundreds of VOCs are found in the urine of a healthy person^1,26^. Since the only so far available method for identifying signs of pathology in VOC samples is a comparison of the data from a healthy and a sick individual, the presence of a large number of compounds makes it extremely difficult to search for specific biomarkers of a disease. At the same time, trained animals can solve this problem. Apparently, they are analysing the "olfactory image" of the disease, consisting of many separate traits. Over the past two decades, significant amount of data have been obtained on trained animals that can successfully distinguish organisms with various diseases and injuries from healthy ones by scent^13–15,23,27–31^.

The search for approaches to solving this problem requires a disease model, which will significantly reduce the non-disease-related differences in the VOC spectra of excreta from sick and healthy organisms and will allow observing the development of the disease and associated changes in the VOC spectrum in a limited period of time.

We chose hepatocarcinoma since the search for biomarkers and the creation of non-invasive screening are especially relevant for diseases for which the effectiveness of the cure is highly dependent on early diagnosis. Research results show that early diagnosisis critical for patient survival and effective treatment of malignant tumours^32^. Therefore, the search for "olfactory images" of these diseases is one of the promising directions of research intovery early symptoms of malignant growth.

It is also known that cancer is a complex disease and, in addition to the actual malignant growth, a sick organism is distinguished from a healthy one by many other processes, including inflammation, angiogenesis, tissue necrosis, etc.^33^. Each of these processes is associated with significant metabolic disorders that change the VOC spectrum, forming a unique "olfactory image" not only of a specific disease but, probably, of each stage of its development.

As tools for comparing the "olfactory images" of sick and healthy animals, we used trained dogs and mass spectrometric analysis of VOCs on a purpose-modified laser mass spectrometer. While some VOC complexes are indeed associated with tumour progression and reflect the metabolism of malignant cells, it can be assumed that with malignant tumour growth associated with an increase in intralesion heterogeneity of cells and their subsequent evolution and selection, the spectrum of secreted substances will change. These changes, apparently, can be registered with the help of "biodetectors" (trained biosensor dogs), and with a mass spectrometer.

For a reliable comparison of the results of detection using a dog and a mass spectrometer, it is necessary to provide similar experimental conditions in both cases. This requires the use of a mass spectrometer operating without sample preparation and recording VOCs in air at room temperature. The main distinguishing feature of the device is the method developed by the authors for ionization of VOCs by vacuum ultraviolet radiation, emitted by the laser plasma generated on a metal target (atmospheric pressure laser plasma ionization, APLPI)^34,35^. To identify individuals with transplanted tumour tissue, we used a probabilistic assessment method based on a limited amount of information obtained from VOC analysis. This is based on the comparison of "olfactory images"—samples of VOC spectra from healthy and ill patients and, in principle, does not require the isolation of specific marker compounds, in the same way as happens when using trained dogs.

The aim of this study was to compare the efficiency of separating sick animals from controls using trained biosensor dogs and the new mass spectrometry method at different stages of tumor growth. We aimed to determine whether the stage of tumor growth affects the success of sick organism detection using dogs and mass spectrometry express analysis, and to compare the results of separating sick animals at different stages of tumor growth using trained dogs and instrumental analysis.

## Materials and methods

### Mouse model of hepatocarcinoma

Male hybrid mice BDF-f1 (DBA2 × C57Bl/6) served as a cancer experimental model. Tumour tissue (100 mg) suspension in 0.5 mL of saline (0.9%) was transplanted subcutaneously to 2-to 4-month-old hybrid males. We employed a transplantable hepatic tumour strain H33 obtained in Blokhin Cancer Research Institute, Institute of Carcinogenesis, from tumours previously induced in the similar hybrid male BDF-f1 mice with a single intraperitoneal injection of 90 mg/kg dose of diethylnitrosamine^36^.

Three types of controls were used: intact male mice of the same strain, males injected subcutaneously with 0.5 mL of saline (0.9%) at the same time as tumour was transplanted, and males with transplanted healthy liver tissue (tissue (100 mg) suspension in 0.5 ml of 0.9% saline) at the same time as tumour was transplanted.

Independently of each other, two groups of mice were used for experiments with dogs and mass spectrometric studies. Since experiments with dogs require a large number of urine samples, and we did not manage to collect urine every day from the strain we used, a group of 265 2-month-old mice was used. To collect samples, we used 100 mice with subcutaneously transplanted hepatocarcinoma and 165 control mice, of which 100 mice were inoculated with healthy liver tissue, 53 mice were injected with saline, and 12 were intact mice (Table 1).

**Table 1:** The composition of the groups of mice and the number of samples collected for the dog experiment.

	Intact mice	Mice injected with saline	Mice with transplanted healthy liver tissue	Mice with transplanted hepatocarcinoma
Number of urine donor mice	12	53	100	100
Donors' age (days)	60–70	60–70	60–70	60–70
Average weight of donors (g)	24.7	24.2	24.8	25.5
Number of urine portions collected	142	695	1547	1469

For mass spectrometric studies, a group of 30 4-month-old mice was used: 10 with transplanted hepatocarcinoma and 20 control mice (10 with transplanted healthy liver tissue and 10 intact) (Table 2).

**Table 2:** The composition of the groups of mice and the number of samples collected for the MS experiment.

	Intact mice	Mice with transplanted healthy liver tissue	Mice with transplanted hepatocarcinoma
Number of urine donor mice	10	10	10
Donors' age (days)	120–130	120–130	120–130
Average weight of donors (g)	25.8	26.4	26.9
Number of urine portions collected	41	40	42

To assess daily changes in the mass of the transplanted tissue, as well as for subsequent histological studies, every day after collecting urine, 20 individuals from the first group of 2-month-old mice were euthanaised: 10 with transplanted hepatocarcinoma and 10 controls with transplanted healthy liver tissue. The transplanted tissue was removed from the animals and weighed, and then the tumour tissue and healthy liver tissue were fixed in preparations.

Mice with the transplanted tumour tissue were euthanized when it appeared that the tumour burden induced discomfort. All animals were kept in the same conditions (ten animals each in standard plastic cages with a bed of wood shavings, in a room with a controlled temperature (21 °C) and with a 12:12-h light:dark cycle).

The experimental procedures were approved by the local ethical committee of the Institute for Information Transmission Problems of the Russian Academy of Sciences (Protocol No. 1 of November 20, 2017). All methods were performed in accordance with the relevant guidelines and regulations of The Declaration of Helsinki and National Institutes of Health Guide for Care and Use of Laboratory Animals. All experiments were conducted in accordance with the ARRIVE guidelines and were performed in accordance with the relevant guidelines and regulations.

### Urine collection

In mice, urine was collected from days 1 to 8 after tissue transplantation (from injection to the stage when the tumourwas visible to the naked eye through the skin in all mice) (Table 1, Table 2).

In the mice used for this study, it was impossible to collect urine in a standard way, holding them by the scruff, so urine samples were collected using special plastic cages with a mesh bottom in which the mice were kept until urination, but no more than for 2 h. The caged mice were monitored continuously, and urine was collected immediately after excretion, making sure that neither subject's body parts nor faeces could enter the sample tube.

Even using this method of urine collection, it was not always possible to collect samples from all mice, and in a group of 30 mice, samples of which were intended for mass spectrometric examination, it was not completely possible to collect urine on day 6 after tissue transplantation. Using an automatic dispenser, urine was divided into portions of 60 μL and transferred for storage into microcentrifuge tubes, which were immediately frozen at − 23 °C and thawed immediately before the experiment.

### Experiments with dogs

For the experiments we used the match-to-sample-likeprotocolemployed in forensic odorology and scientific research^23,37,38^ this method is described in detail by Rodionova et al.^23^. In general terms, the procedure consisted of the dog being encouraged to sniff a tube containing a urine sample from a donor with transplanted hepatocarcinoma (odour to search, initial sample), and then prompted to choose from 12 similar tubes in a lineup, among which was a sample of another donor with transplanted hepatocarcinoma (test sample). To compare the urine samples, specially trained biosensor dogs (jackal-dog hybrids) were used from the canine department of the Cynological Division of Aviation Security Service, Aeroflot, Russian Airlines ". In total, five adult dogs (three males and two females), aged 5–10 years, were used as sensors.

In an experimental room measuring 4 × 4 m, a temperature of 18–20 °C and a humidity of at least 35% were maintained. Twelve points were drawn on the floor of the room to form a circle. The points were located at a distance of 1 m from one another; 60 μL of mouse urine in an open microcentrifuge tube was placed on the bottom of a cleanly washed 0.5 L glass jar so that the dog, while sniffing, could not touch the source of the odour.

In addition to the test sample, we set ten or eleven control samples. The control samples were the same in most lineups, the urine of mice with transplanted liver tissue (75 lineups). Sometimes several urine samples of mice with transplanted liver tissue were replaced with other control samples: urine samples of mice injected withsaline (22 lineups) and/or urine samples of intact mice (4 lineups).The initial sample was presented to the dog for 30 s and then a series of samples was presented after 1–2 min.

Samples were placed in a circle in random order. All urine samples in the same circle were collected on the same day from different animals and presented to only one dog. Presenting such a lineup to a dog was considered as one experiment. The experiments were conducted in a double-blind manner, i.e. neither the instructor leading the dog nor the observers in the room knew the location of the samples.

When a urine sample from an animal with transplanted tumour tissue was found, the dogs sat down in front of the jar and barked. We recorded that the dog made the right choice (a true-positive response) if it found a jar containing mouse urine with injected tumour tissue. If the dog sat down before the control urine sample, we recorded this choice as erroneous (false-positive response). In the case when the dog sniffed out all the jars and didn't choose any or gave out three false (false-positive) signals, we recorded that the animal did not find the test sample. With urine samples collected on every day of tumour development, 10–15 experiments were carried out to identify animals with transplanted tumour tissue using detection dogs (99 experiments in total). All experiments were recorded on paper and on video.

### Mass spectrometry studies

**Figure 1:**
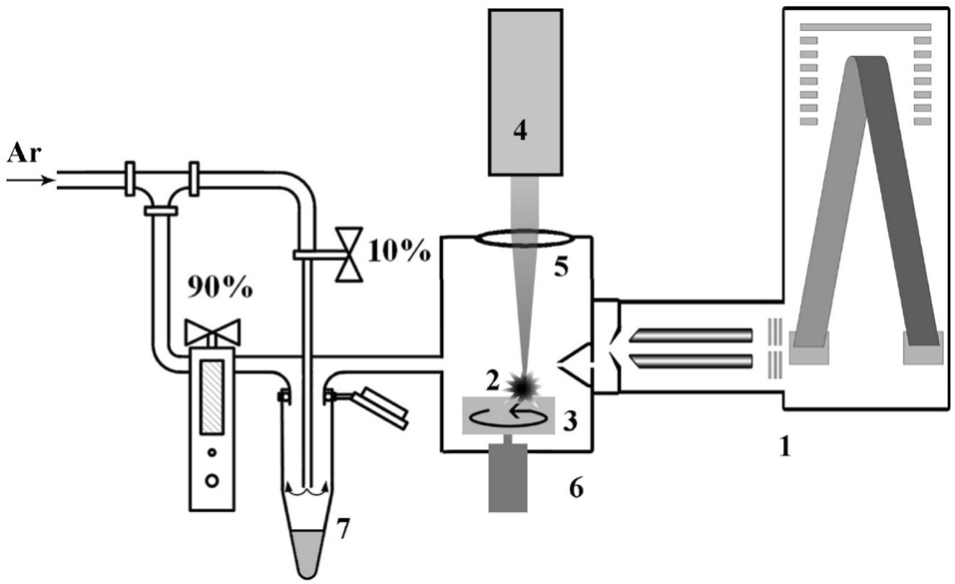
Schematic diagram of the laser mass spectrometer: 1—reflectron time-of-flight mass analyzer (m/∆m = 5000), 2—laser plasma, 3—rotating metal target, 4—diode-pumped Nd:YAG laser (pulse duration 0.5 ns, pulse energy 400 μJ), 5—focusing lens, 6—ionization chamber, 7—microcentrifuge tube.

Mass spectrometric studies were carried out using a unique setup developed by the authors of the study (Fig. 1). The description of the setup has been given in details in^35,39^. It differs from commercial devices in (i) the way VOCs are ionized, (ii) the absence of a hot capillary at the inlet of the device, and (iii) an appliance for introducing VOCs from a urine sample in the as-is state at room temperature. The main feature of the mass spectrometer used in the experiment is the use of vacuum ultraviolet radiation from the laser plasma, created on a metal target by pulsed Nd:YAG laser radiation, for ionization of VOCs at atmospheric pressure.

A microcentrifuge tube (7, Fig. 1) with a urine sample was installed on a sealed pneumatic connector of the inlet line of the ionization chamber and was purged with a flow (~ 6 cm^3^/ s) of pure argon (99.995). Urine VOCs were transferred in a carrier gas stream into a sealed chamber where they were ionized. To eliminate the ingress of ambient atmospheric air into the chamber when changing tubes, an overpressure of 30 torr was maintained in the chamber. The VOC mass spectrum of each sample was recorded for about 2 min, of which about 30 s were required to replace the air with argon.

A urine sample in a microcentrifuge tube and all elements of the ionization chamber were at room temperature, which made it possible to compare with the experiments conducted with dogs.

Before the measurements, the frozen samples were thawed at room temperature for 60 min and, without additional preparation, were poured into microcentrifuge tubes in portions of 20 μL. The studies were performed blindly with a random sample choice. The operator of the mass spectrometer knew nothing about the sample under study, except for its nominal number.

Our experiments did not use chromatographic separation and fragmentation to identify individual VOC components. The mass spectrum was analyzed in its entirety, as an "image" containing information about a possible disease of the organism. This approach, reminiscent of the actions of a trained dog, provides a dramatic reduction in analysis time, but the amount of information obtained about the composition of VOCs in our experiments is limited by the resolution of the mass spectrometer. There was no significant information in mass spectra with m/z higher than 500 corresponding to ions of VOCs. For this reason, the mass spectra were truncated to the range of m/z 18–500.

### Result representation

The dynamics of changes in the transplanted tissue is shown in Fig. 3 by scatter diagrams of the measured values of tissue mass and graphs of their median values per day in two samples, for mice with transplanted tumour tissue and mice with transplanted healthy liver tissue.

The dynamics of the dogs' reaction are represented by the proportions of true positive and false positive choices relative to all reactions in all experiments conducted with urine samples collected on a certain day (1–8) after tissue transplantation, with a 95% confidence interval.

Based on the tasks of the study, we focused on the general analysis of differences in the mass spectra of control individuals and individuals with transplanted tumour tissue. These differences were recognised using principal component analysis.

**Figure 2:**
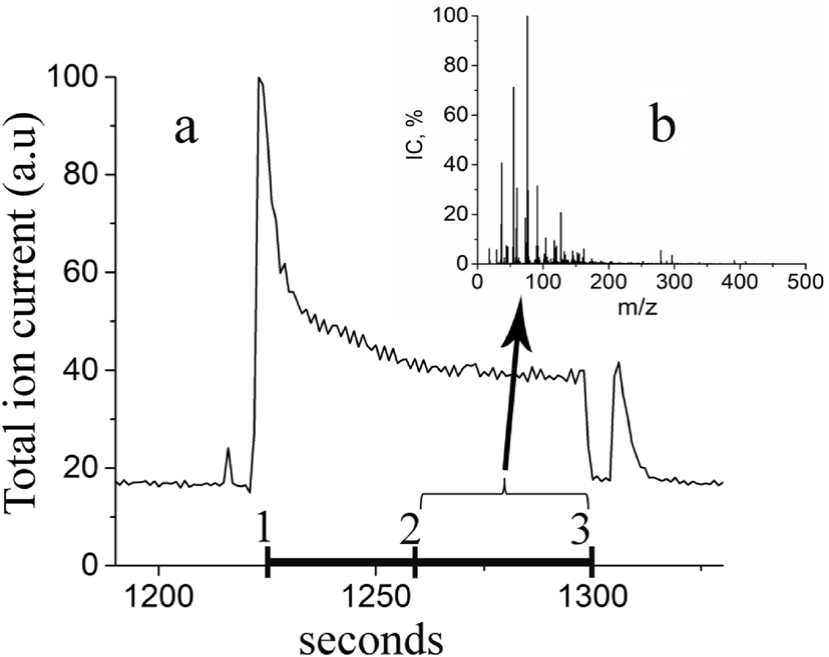
(**a**) Time dependence of the total ion current (sample 8379) and (**b**) cumulative mass spectrum of the sample.

Figure 2 shows the time dependence of the total ion current of the mass spectrometer and a typical cumulative mass spectrum of urine VOCs. The interval for recording the mass spectrum of a sample is highlighted on the time axis with a bold segment marked with numbers 1, 2, and 3. The test tube is set at point 1. In the initial sections 1–2, a high rate of change in the ion current is observed, due to the displacement of air from the test tube with the sample. After 30 s, the ion current is stabilized and the summation of 40 single mass spectra obtained from one sample in 40 s is carried out in the range of m/z 18–500 (sections 2–3).

In mass spectrometric studies, a total of 123 urine samples were analysed, obtained within 8 days after inoculation of healthy liver tissue and hepatocarcinoma (except for day six, when it was not possible to collect mouse urine).

Since it was not always possible to collect urine from all 30 animals, the number of urine samples obtained on some days was insufficient, therefore, based on our data on the development of transplanted tumour tissue, the spectra of collected urine samples were combined into three groups for principal component analysis: the first group includes the mass spectra of samples for the 1st–3rd days, the second group for 4th–5th days, the third group for 7th–8th days).

The groups of mass spectra were imported into the Jupyter Notebook of a Python program and the PCA function was applied with the help of the Scikit-learn library. The data plotted in the first two components create characteristic fields for each group. Each mass spectrum of a specific sample corresponds to one point in the principal component space. The division into clusters in the coordinates of the first two principal components corresponding to three groups of animals was conducted using the logistical regression algorithm.

## Results

### Change in the mass of transplanted tissue

**Figure 3:**
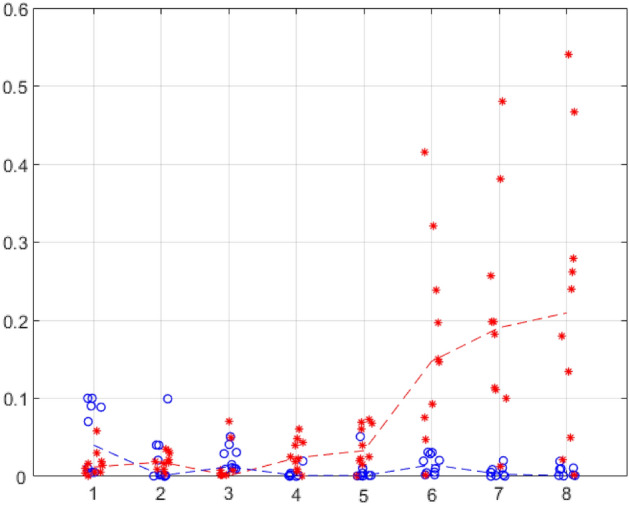
Changes in the tissue mass of hepatocarcinoma H33 (red asterisks) and healthy liver tissue (blue circles) by days after transplantation. The horizontal axis shows days after transplantation, and the vertical axis shows values of the mass of the altered tissue ("tumour") in grams. For clarity, the medians of the same type of mass measurements are connected by dashed lines.

Figure 3 shows the experimentally measured values of tissue mass and graphs of their median values by day in two samples, for mice with transplanted hepatocarcinoma and mice with transplanted healthy liver tissue. It shows that the mass of the transplanted tumour tissue, like the mass of the transplanted healthy liver tissue, slightly decreases in the first 3 days. On the 4th day, the mass of the transplanted healthy liver tissue becomes close to zero, and the mass of the tumour tissue begins to grow, and by the 6th day, it is several times greater than the volume of the tissue initially transplanted.

### Experiments with biosensor dogs

Table 3 shows how many times in the conducted experiments the dogs reacted positively to various control samples (false positive responses) and how many to test samples (true positive responses).

**Table 3:** Dogs' responses to control and test samples.

Sample types	Number of the samples	Number (percentage) of dog responses to the samples
Urine from intact mice	38	3 (7.9%)
Urine of mice injected with saline	205	22 (10.7%)
Urine of mice with transplanted healthy liver tissue	760	127 (16.7%)
Urine of mice with transplanted hepatocarcinoma	99	68 (68.7%)

**Figure 4:**
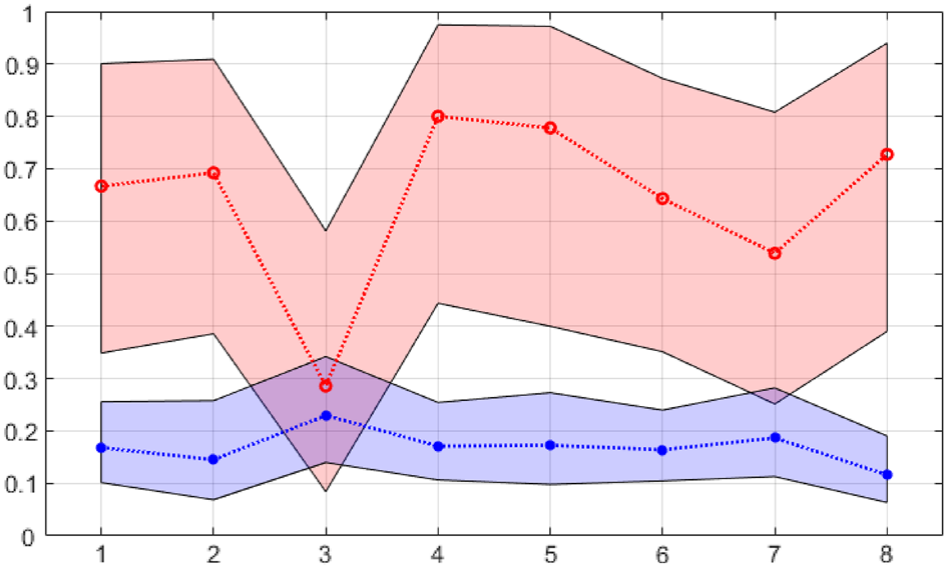
Relative frequency of true positive and false positive responses of dogs to urine samples collected by day. Red and blue markers show the proportions of true positive and false positive reactions of dogs to urine samples, respectively. The shaded areas correspond to the 95% confidence interval for each of the 8 days of study.

It can be seen that, although dogs respond to samples from mice with transplanted hepatocarcinoma significantly more often than to any control samples, they still respond to only slightly more often than half of the cases. We analysed how the reactions of dogs to test samples depend on the time elapsed since tissue transplantation (Table 4, Fig. 4).

**Table 4:** The number of urine samples from mice with transplanted tissue for each of the eight days after transplantation.

Number of days after tissue transplantation	Number of urine samples from mice with transplanted healthy liver tissue	Number (percentage) of false positives in dogs	Number of urine samples from mice with transplanted tumour tissue	Number (percentage) of true positive dog responses
1	101	17 (16.8%)	12	8 (66.7%)
2	62	9 (14.5%)	13	9 (62.2%)
3	74	17 (22.9%)	15	5 (33.3%)
4	111	19 (17.1%)	10	8 (80%)
5	81	14 (17.2%)	15	11 (73.3%)
6	128	21 (16.4%)	14	9 (64.3%)
7	91	17 (18.7%)	14	8 (54.1%)
8	112	13 (11.6%)	13	10 (76.9%)

On the first and second day of growth of the transplanted hepatocarcinoma tissue, dogs successfully distinguish mice with transplanted tumour tissue from mice with transplanted healthy liver tissue by the scent of the urine; however, on the third day of tumour growth, the number of dogs' reactions to urine samples from mice with transplanted tumour tissue drops drastically, that is, dogs no longer distinguish between urine samples from animals with transplanted hepatocarcinoma and healthy liver tissue. From the fourth day of tumour tissue development, detection of diseased mice does not cause difficulties for dogs, and although the detection rate is not always the same, on average it is always above 50%.

### Mass spectrometry studies

**Figure 5:**
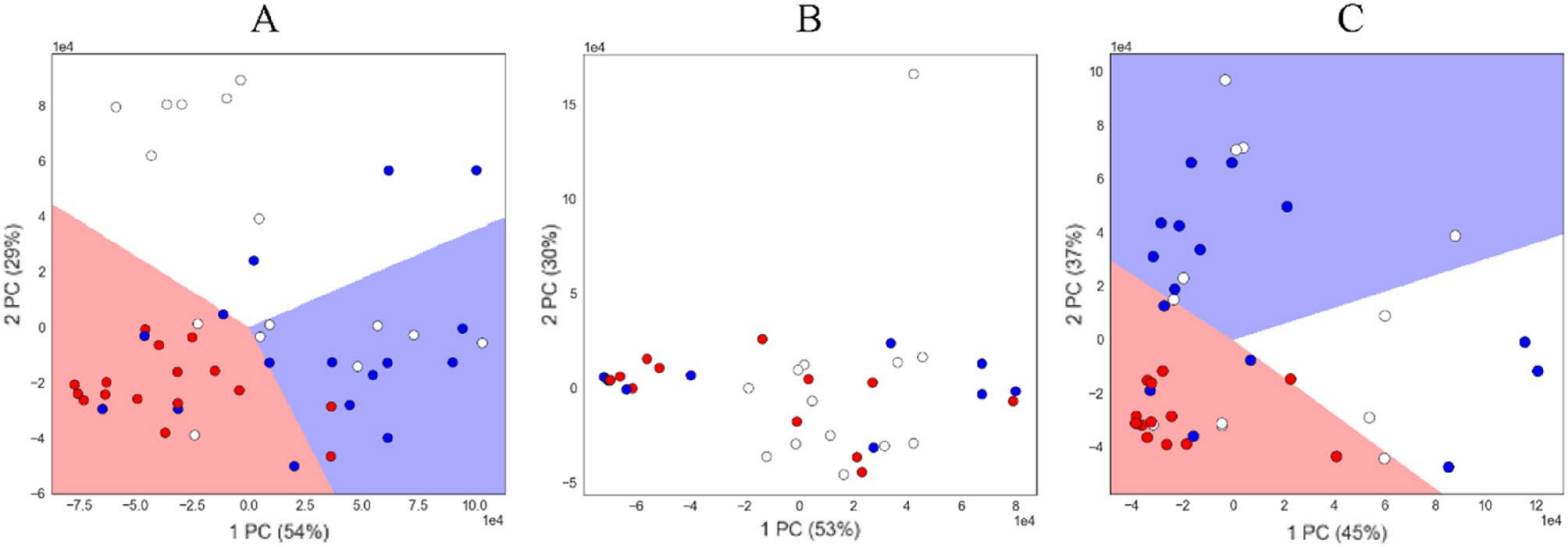
Results of applying PCA to mass spectra for three groups by day after tissue transplantation: (**A**) 1–3 day, (**B**) 4–5 days, (**C**) 7–8 days. White circles are urine samples from intact mice, blue circles are urine samples from mice with transplanted healthy liver tissue, red circles are urine samples from mice with transplanted hepatocarcinoma tissue. The urine samples spreading over the days after tissue transplantation are presented in Table 3.

In mass spectrometric studies, we used urine samples from control mice (intact and with transplanted healthy liver tissue), and mice with transplanted hepatocarcinoma tissue. Figure 5 shows the results obtained in the coordinates of the first two principal components, and Table 2 shows the distribution of samples by day after tissue transplantation.

The results show that urine samples from mice with transplanted hepatocarcinoma are steadily excreted on days 1–3 and 7–8 after tissue transplantation. On days 4–5, there is no isolation of any type of samples, what is close to what is observed in the experiments with dogs (Fig. 4, Table 5) and is apparently associated with a change in the mass of the transplanted hepatocarcinoma tissue.

**Table 5:** Urine samples spreading over the days after tissue transplantation.

Days after tissue transplantation	Total number of urine samples	Number of urine samples from mice with transplanted tumour tissue	Number of urine samples from mice with transplanted healthy liver tissue	Number of urine samples from intact mice
1–3	47	16	15	16
4–5	35	12	10	13
7–8	41	14	15	12

## Discussion

In the present study, we tested whether the growth stage of tumour tissue affects the unique chemical characteristics of the urine of mice and to what extent the “olfactory image of disease” perceived by dogs matches the “disease image” recorded by the mass spectrometer.

As a model, we used mice, with transplanted hepatocarcinoma tissue. In this case, the differences in the genetics of the "test" and control animals, in their diet, age, everything can alter the "olfactory image of the organism", except for the disease, is minimized. In addition, the transplanted tumour tissue makes it possible to study the change in the “olfactory image” of the disease at different stages of tumour growth in the body. An exactly known onset of the disease, and the rapid development of the tumour, which literally changes by the day, makes it possible to compare the odour of the same organisms at different stages of the disease. The development of the disease can be roughly estimated by the amount of tumour tissue.

In our experiments, both the success of dogs and the success of laser mass spectrometry in detection of a sick organism among healthy ones are in good agreement with the dynamics of the development of the transplanted tumour tissue. From the first to the third day, the main process taking place at the site of localization of the transplanted tissue is a decrease in its relative mass, both in most mice with tumour tissue and in most mice with healthy liver tissue. From about the third day, everything changes, i.e., the amount of transplanted healthy liver tissue continues to decrease, while the amount of tumour tissue begins to increase rapidly. It seems that on day 3, the processes in the experiment and control reach the greatest similarity: the immune system reacts to foreign cells, which is reflected in the reaction of the dogs. The activation of tumour proliferation on the 5th day after tissue transplantation coincides with an increase in the sensitivity of dogs to the odour of the disease. It can be concluded that it is this process that is the source of metabolites that mark the diseased organism at this stage of tumour development. However, a "critical mass" of malignant cells is needed to alter the VOC spectrum in urine.

The experiment with mass spectra showed that while on days 1–3 we see the division of urine samples into three groups (intact animals, animals with transplanted tumour tissue, and animals with transplanted healthy liver tissue), on days 4–5 there are no differences between the spectra, but on the 7–8th day only a group of animals with translanted tumour tissue stands out clearly, while all control animals, both intact and animals with transplanted healthy liver tissue, effectively form one group.

This also coincides well with the dynamics of changes in the mass of the transplanted healthy liver tissue: On days 1–3, there is a sharp decrease in its mass, that is, there is an active immune response of the body to the presence of foreign tissue, while on days 6–8 this tissue almost disappears, the immune reaction ceases and the "olfactory image" of mice with transplanted healthy liver tissue becomes similar to the "olfactory image" of intact animals.

It is noteworthy that the two groups of control animals are also distinguished by physical trauma of injection, which, as we have shown earlier^17^, also increases the differences in the "olfactory images" of the two groups of control animals, but this difference gradually decreases with healing of the injection wound.

There are some differences in the results obtained from experiments with dogs and using mass spectrometry. While on the 3rd day after transplantation, the dogs cease to distinguish between urine samples from mice with transplantedtumour tissue and healthy liver tissue, we observe no differences in mass spectra on days 4–5. We believe this is due to the use of different groups of mice in these experiments. The fact is that, as can be seen from Fig. 1, the dynamics of the development of tumour tissue is not the same in different individuals, and probably depends not only on the individual but also on how the tumour tissue developed before inoculation. Figure 1 shows that in two mice on the 3rd day after inoculation, the mass of tumour tissue is significantly greater than in the others. This is possible for two reasons, either by the 3rd day there is still a lot of transplanted tissue, or, on the contrary, the selection of new tumour cells and tumour growth for some reason in these mice is more active than in others. The same figure shows great differences in the further change in tumour mass in different mice. This is consistent with the well-known fact that global rearrangements of the body's metabolic pathways in the case of the development of each tumour have individual characteristics since malignant growth is associated with gene damage and subsequent cancer evolution, e.g., with clonal selection of malignant tumour cells^40^.

As the mass of tumour tissue on the fourth day after transplantation increases insignificantly (Fig. 3), and the number of correct choices of urine samples of a sick organism by dogs at the same time more than doubles, we believe that the success of dogs' recognition of a "sick" organism is associated not with the mass of tumour tissue, but with the activity of tumour proliferation. Metabolites marking processes specific to tumour growth are excreted in the urine, and since the odour presentation technique ensures that biosensors do not have direct contact with odour sources, it can be concluded that these are VOCs, which may be potential markers of the initial stages of tumour development. The experiment with dogs was carried out simultaneously with the measurement of the tumour mass in one group of mice. For mass spectrometry, a different group was used, in which, possibly, similar processes proceeded at a different rate. However, we observe essentially the same dynamics in these two groups, only slightly shifted in time.

The mechanisms of occurrence and release of VOC spectra, the "scent of disease", are poorly understood. For some diseases, the mechanism underlying the change in body odour is obvious and highly non-specific. For example, diseases associated with GI disorders alter the odour of faeces^41^. Microorganisms release their metabolites into the host's body, and these volatiles are excreted in respiration, urine, faeces and sweat. Clinicians know odours specific to specific infections, and VOC profiles can potentially be used as biomarkers of infectious diseases^42^. For example, the faeces of patients with cholera have a characteristic sweetish odour. Based on the analysis of VOCs in fecal samples from patients with cholera, dimethyl disulphide and terpineol have been identified as potential biomarkers^24^.

One potential pathway that causes the odour of secretions to change during illness is through the immune system. It is known that vaccination can cause significant changes in body odour, and volatile metabolites can arise at different stages of complex processes of innate or adaptive immunity^14^. Long-term studies have shown that the major histocompatibility complex (MHC) is closely related to VOCs secreted by the body^43,44^. The authors concluded that the volatile signals detected by trained animals can be influenced by lipases, cytokines, and complementary protein complexes, singly or in combination. The accumulated data indicate that practically every disturbance in the animal body leads to a change in the VOC spectrum. Changes in urine odour associated with immunization^14^, infectious diseases^13^, chronic disease^15^, and brain damage^16^ have been described in animal experiments. These studies provide evidence of characteristic changes associated with various metabolic pathways in the body, giving hope that volatile metabolites may specifically mark many diseases.

Selective cell growth, characteristic of tumour development, their proliferative advantage, reprogramming of energy metabolism, changes in the stress response, favorable for overall cell survival, tumour vascularization, invasion, metastasis, specific tumour microenvironment or the formation of premetastatic niches, immune modulation and other processes are associated with significant rearrangements of the metabolism not only of tumour cells but also cells of the immune system, tumour microenvironment, etc.^33,45^. These processes are based on the instability of the tumour cell genome, which generates the genetic diversity of cells^33^. All these features are associated with the restructuring of metabolism, which must affect the composition of urine, the composition of its volatile components and, consequently, smell^46^.

A large number of metabolic rearrangements accompany even not very significant cellular events in the body, indicating not only that the number of possible combinations of odourants in the urine is enormous, but also that the likelihood of the existence of a unique volatile substance associated with malignant growth is almost excluded. Unique VOC markers are probably only characteristic of bacterial infections. Moreover, the VOC spectra of sick and healthy (control) organisms differ, most likely, not in the composition, but in the ratios of the same substances.

Our data suggest that both dogs and our proposed method of laser mass spectrometry are capable of detecting both the entire spectrum of VOCs associated with the disease and minor changes in this spectrum during its course. Using "model patients" (mice), comparing the stages of development of the disease, the response of dogs, histological and physiological analysis of the processes occurring in the body with the VOC spectra obtained using modern analytical methods, over time, it will be possible to identify metabolic pathways responsible for the changes in odour and the diagnostic potential of VOC spectra. Studies of the composition of VOCs in the early stages of tumour growth are useful for finding early diagnostic markers of malignant growth and may also shed light on the mechanisms underlying changes in the VOC spectrum during malignant growth.
